# Nitrate of Silver in Membranous Croup

**Published:** 1849-07

**Authors:** V. M. Satterlee

**Affiliations:** Green Bay, Wis.


### ARTICLE III.

*Nitrate of Silver in Membranous Croup.*—By V. M. SATTERLEE, M. D., of Green Bay, Wis.

In September last I was requested to visit Miss M. G., æt 9 years with "*Cynanch trachealis.*"

Found her with face flushed and swollen; eyes protrubed; laborious respiration, giving rise to a frightful hissing noise; pulse 115 a minute.

Gave an emetic immediately, with temporary relief; applied mustard, then dilute· nitric acid to the throat, and prescribed such other remedies as the circumstances of the case seemed to demand.

Breathing became louder and might be heard all over the house, symptoms more urgent, and patient fast sinking under the disease.

I then detemined, as a last resort, to use strong solution of nitras argenti, which was prepared, and a piece of soft sponge well saturated with it, was introduced low down in the trachea. In less than half an hour from this time the breathing became much easier, and considerable expectoration of ropy matter was obtained, which gave instant relief, and within one hour from the time of using the caustic, a piece of *false membrane* was thrown off, being an inch long, hollow, and tube like. The effect was immediate. Her breathing, so distressingly performed a short time before, was now nearly natural, and she could speak distinctly, which she could not do for three days previously.

